# Latent mitochondrial DNA deletion mutations drive muscle fiber loss at old age

**DOI:** 10.1111/acel.12520

**Published:** 2016-08-25

**Authors:** Allen Herbst, Jonathan Wanagat, Nashwa Cheema, Kevin Widjaja, Debbie McKenzie, Judd M. Aiken

**Affiliations:** ^1^ Centre for Prions and Protein Folding DiseasesDepartment of Agricultural, Food and Nutritional SciencesUniversity of AlbertaEdmontonABCanada; ^2^ Division of GeriatricsDepartment of MedicineDavid Geffen School of MedicineUniversity of CaliforniaLos AngelesCAUSA; ^3^ Centre for Prions and Protein Folding DiseasesDepartment of Biological SciencesUniversity of AlbertaEdmontonABCanada

**Keywords:** aging, mitochondrial DNA deletion mutation, sarcopenia, muscle

## Abstract

With age, somatically derived mitochondrial DNA (mtDNA) deletion mutations arise in many tissues and species. In skeletal muscle, deletion mutations clonally accumulate along the length of individual fibers. At high intrafiber abundances, these mutations disrupt individual cell respiration and are linked to the activation of apoptosis, intrafiber atrophy, breakage, and necrosis, contributing to fiber loss. This sequence of molecular and cellular events suggests a putative mechanism for the permanent loss of muscle fibers with age. To test whether mtDNA deletion mutation accumulation is a significant contributor to the fiber loss observed in aging muscle, we pharmacologically induced deletion mutation accumulation. We observed a 1200% increase in mtDNA deletion mutation‐containing electron transport chain‐deficient muscle fibers, an 18% decrease in muscle fiber number and 22% worsening of muscle mass loss. These data affirm the hypothesized role for mtDNA deletion mutation in the etiology of muscle fiber loss at old age.

## Introduction

Sarcopenia is an increasingly prevalent and disabling consequence of aging, and the lack of a clear etiology is a critical barrier to developing treatments. This functional decline is a defining characteristic of the geriatric syndrome of frailty, which encompasses weight loss, muscle weakness, and slowed gait. A primary contributor to frailty is sarcopenia—the inevitable age‐related process that results in the loss of skeletal muscle mass and function (Rosenberg, 1997). In humans, sarcopenia begins at ~25 years of age, approaches a 10% loss of muscle mass by age 50 and a 50% loss by the eighth decade (Lexell *et al*., 1983; Goodpaster *et al*., 2006). Muscle mass decline in humans has been attributed to both fiber loss and fiber atrophy (Lexell *et al*., 1986; Klein *et al*., 2003). The magnitude of this fiber loss in humans is estimated at ~25 muscle fibers per day at age 80 (Lexell *et al*., 1986). In the F344xBN F1 hybrid rat, muscle mass and fiber loss begins at ~30 months of age and ~20 fibers are lost per day (Wanagat *et al*., 2001; Lushaj *et al*., 2008), paralleling the human data.

The mechanisms that drive muscle fiber loss are mediated by apoptosis and necrosis in aged skeletal muscle (Dirks & Leeuwenburgh, 2004; Marzetti *et al*., 2010). Down‐regulation of the apoptotic pathway reduces muscle mass loss and improves function in aged animals (Dupont‐Versteegden, 2005). In aging human muscle, increased levels of apoptosis have been detected from biopsies (Malmgren *et al*., 2001; Whitman *et al*., 2005). Cellular necrosis is another mechanism of muscle fiber loss and is readily observed in aging rat muscle (Fujisawa, 1974). Electron transport chain (ETC) deficiencies trigger muscle fiber apoptosis and necrosis in aging skeletal muscle (Cheema *et al*., 2015).

Electron transport chain (ETC) abnormal muscle fibers arise from a very specific molecular event, the intracellular, clonal accumulation of mitochondrial DNA deletion mutations. Mammalian mitochondria contain their own ~16‐kb circular DNA genome that, together with nuclear components, encodes the proteins in the ETC. Muscle mitochondria exist as an extensive interconnected reticulum and cross sections of muscle contain thousands of mitochondrial genomes (Bakeeva *et al*., 1978; Ogata & Yamasaki, 1997; Herbst *et al*., 2007; Mishra *et al*., 2015). When mtDNA deletion mutation‐containing genomes accumulate to high levels, the defective genomes interfere with the normal transcription and translation of the mitochondrially encoded components of the ETC (NADH dehydrogenase, cytochrome b, cytochrome c oxidase‐COX, ATP synthase). High levels of deletion‐containing mtDNA genomes are localized to the ETC abnormal region of individual fibers (Müller‐Höcker *et al*., 1993; Cao *et al*., 2001; Wanagat *et al*., 2001). Although the specific deletion mutation differs from fiber to fiber, within a single fiber, ETC abnormal regions contain identical mtDNA deletion mutations suggesting that once a mitochondrial deletion mutation occurs, it expands clonally from its point of origin (Cao *et al*., 2001). As multiple copies of the mtDNA exist in the mitochondrial reticulum, both wild‐type and mutant mtDNA coexist in a state of heteroplasmy. The cellular impact of heteroplasmy is largely dependent on the mutant to wild‐type mtDNA ratio. MtDNA deletion mutation abundance approaches 100% of the mitochondrial genomes within the ETC abnormal region of the fiber, and the threshold level for expression of the ETC abnormal phenotype is 90% (Herbst *et al*., 2007). Low abundance mtDNA deletion mutations have been detected in ETC normal fibers and represent a pool of latent mutations that could give rise to respiration deficiency and cell death given sufficient time or appropriate stimulation to accumulate(Pak & Aiken, 2004; Herbst *et al*., 2007).

Gene expression measurements from diverse models of mitochondrial dysfunction and mitochondrial diseases (Alemi *et al*., 2007; Subramaniam *et al*., 2008) and studies from laser microdissected ETC abnormal regions of skeletal muscle fibers (Herbst *et al*., 2013) identified the activation of mitochondrial biogenesis. Beta‐guanidinopropionic acid (GPA), a creatine analogue, induces mitochondrial biogenesis primarily in skeletal muscle (Wiesner *et al*., 1999; Reznick & Shulman, 2006). GPA, by inhibiting the creatine transporter, decreases intracellular creatine, high energy phosphocreatine, and ATP in skeletal muscle (Oudman *et al*., 2013), increasing the AMP/ATP ratio (Bergeron *et al*., 2001). The resulting energy deficit, likely through chronic AMPK activation, initiates mitochondrial biogenesis (Zong *et al*., 2002; Chaturvedi *et al*., 2009; Yang *et al*., 2015). Similarly, creatine deficiency increased mitochondrial mass and respiratory enzyme activity in knockout mice (Schmidt *et al*., 2004). In 28‐month‐old rats, GPA treatment resulted in a twofold increase in skeletal muscle wild‐type mtDNA, indicating mitochondrial biogenesis (Herbst *et al*., 2013).

These observations, taken together, suggest that somatically derived mtDNA deletion mutations clonally expand within individual fibers until a phenotypic threshold is surpassed resulting in the loss of cellular respiration, fiber atrophy, apoptosis, and necrosis with ensuing fiber breakage and loss. We hypothesized that, with advancing age, this process increasingly occurs in other muscle fibers—iteratively and cumulatively leading to the observed fiber loss that contributes to sarcopenia (Wanagat *et al*., 2001; Herbst *et al*., 2007; Cheema *et al*., 2015). To test this hypothesis, we treated aged rats with GPA, inducing latent deletion mutation accumulation and the formation of ETC abnormal fibers. We observe a significant increase in mtDNA deletion mutation frequency and ETC abnormality mediated fiber loss.

## Results

To explore the role of ETC abnormal fibers in muscle fiber loss, we treated 30‐month‐old hybrid rats with 1% beta‐guanidinopropionic acid, compounded in chow, for 4 months. There was a 28% decrease in body mass in GPA‐treated rats. There was no significant difference in brain mass, heart mass (Supporting Information), or mortality between GPA‐treated and control rats.

**Figure 1:**
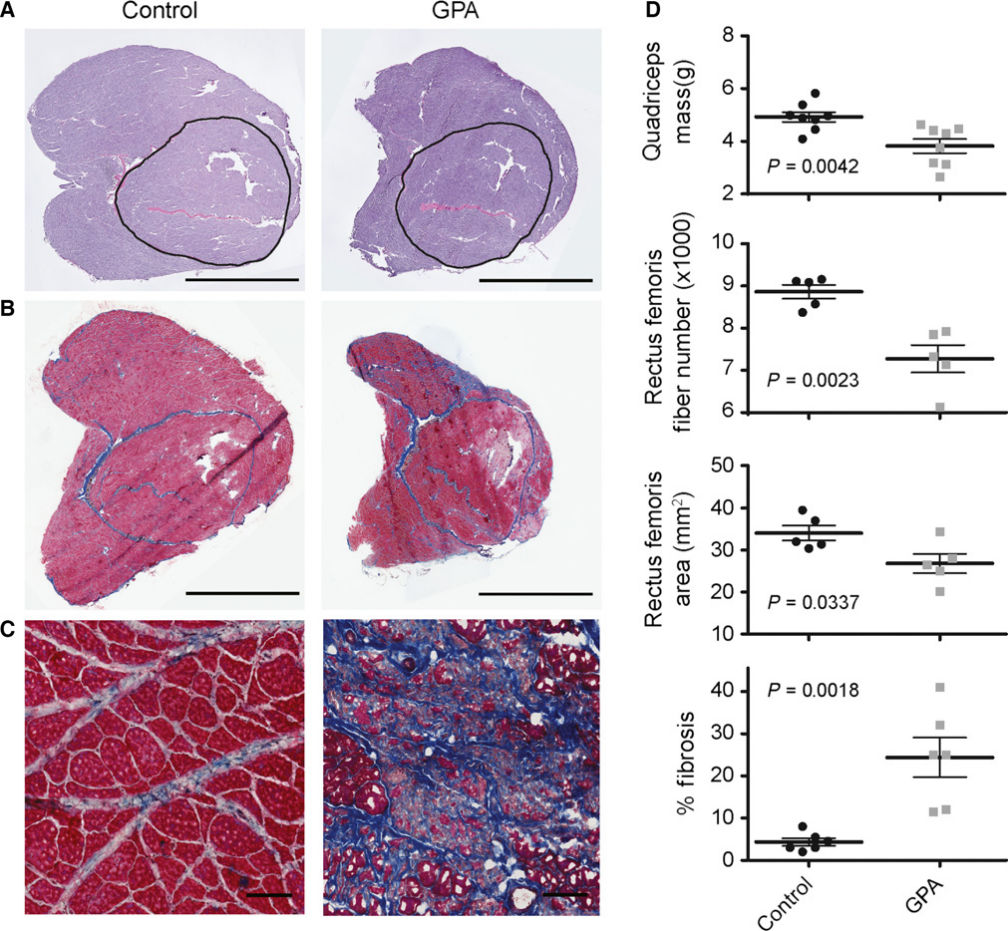
Morphometric data from aged control and GPA‐treated rats. (A) Representative muscle cross‐sectional images stained with hematoxylin and eosin. The bar denotes 5 mm. The encircled area delineates the rectus femoris muscle with cross‐sectional areas of 39.5 and 33.2 mm^2^ for control and GPA treated, respectively. (B) Quadriceps muscle sections stained with Masson's trichrome to identify collagen (blue) deposition. The bar denotes 5 mm. (C) Higher magnification imaging of fibrotic tissue deposited in aged rats. The bar denotes 100 μm. (D) Quadriceps muscle mass, rectus femoris fiber number and cross‐sectional area, and quadriceps fibrotic tissue abundance in control and GPA‐treated aged rats.

GPA treatment reduced quadriceps muscle mass 22% at 34 months of age (Fig. 1A,D). Muscle mass loss was accompanied by a 21% decline in rectus femoris cross‐sectional area and an 18% loss of muscle fibers (1589 fibers on average) in the GPA‐treated rats (Fig. 1A,D). Concomitant with the loss of muscle mass and fibers was an increase in the abundance of excess fibrous connective tissue as detected by Masson's trichrome staining (Fig. 1B–D). In contrast to the typical interfascicular collagen deposition observed in normal muscle aging, we found fibrotic replacement of entire muscle fascicles (Fig. 1C). Muscle deterioration was most prominent in the muscle periphery with sparing in the vastus intermedius and central regions of the lateralis, medialis, and femoris.

**Figure 2:**
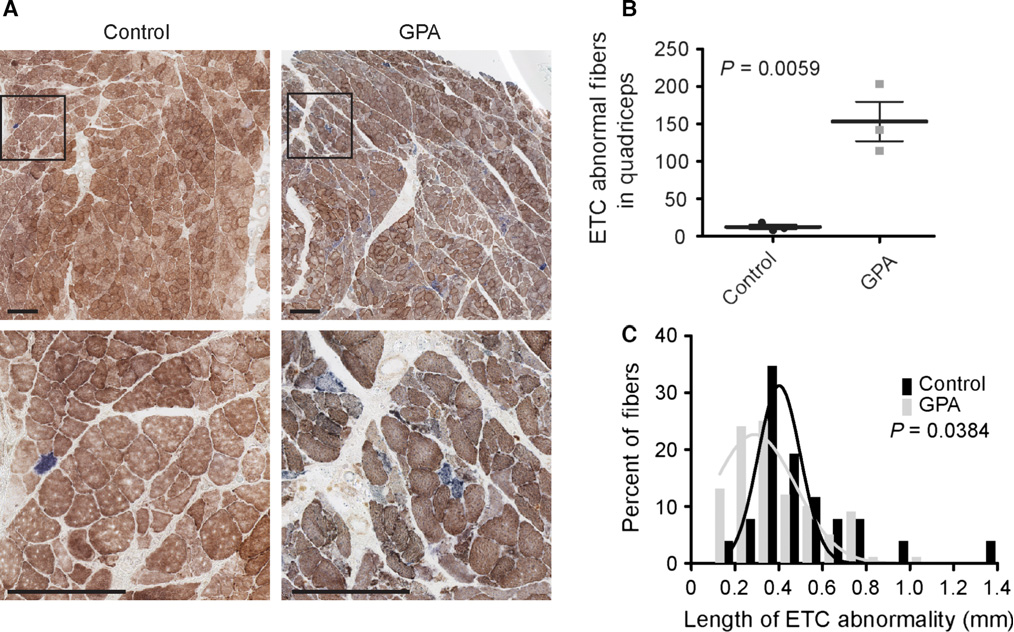
GPA treatment of aged rats results in a 1200% increase in the abundance of segmental ETC abnormal fibers. (A) COX‐negative, SDH hyper‐ETC abnormal muscle fibers are prevalent in GPA‐treated rat muscle. The scale bar in each image is 0.25 mm. (B) ETC abnormality abundance estimates in rectus femoris and quadriceps muscles. (C) GPA treatment decreases ETC abnormal segment length. Frequency distributions are fitted with Gaussian curves using a least squares approach.

To estimate the difference in the tissue burden of ETC abnormal muscle fibers between GPA and control rats, we counted the number of enzymatically abnormal fibers in single muscle cross sections that were histochemically stained for cytochrome c oxidase (COX) and succinate dehydrogenase (SDH) activities. Whereas COX is partially encoded by the mitochondrial genome, SDH is encoded entirely in the nucleus. Individual (COX or SDH) and dual (COX and SDH) staining was used to reveal respiratory‐deficient (COX‐, SDH++) ETC abnormal skeletal muscle fiber segments (Fig. 2A). GPA treatment resulted in a 1,200% increase in the number of ETC abnormal fibers as compared to normally aged control rats within a single muscle section (Fig. 2B). These counts however underestimate the true abundance of ETC abnormal fibers due to their distribution. The volume density of ETC abnormalities (which accounts for the segmental nature and varying length of ETC abnormalities) was estimated by examining 119 randomly selected fibers along their length, through one mm of quadriceps from GPA‐treated rats. By this method, the abundance of ETC abnormal fibers was estimated to be 15.1%. In contrast to normally aged rats, we observed clustering of ETC abnormal fibers, including the appearance of adjacent ETC abnormal fibers, which were not the result of fiber splitting. As observed in normally aged rats, ETC abnormal fibers tended to appear on the exterior surface of the fascicles and some ETC abnormal fibers split into multiple branches. As with collagen deposition, COX‐fibers were most prominent in the muscle periphery. ETC abnormal fibers were not as frequent in the vastus intermedius and more central regions of the lateralis, medialis, and femoris. In addition to increasing the abundance of ETC abnormal fibers, GPA treatment also affected the morphological characteristics of ETC abnormal fibers. The population of ETC abnormal fibers from GPA‐treated rats possessed, on average, shorter ETC abnormal segments. The mean ETC abnormality segment length in control rats was 442 ± 49.0 μm vs. 345 ± 19.4 μm (*P* = 0.0384) following GPA treatment (Fig. 2C).

**Figure 3:**
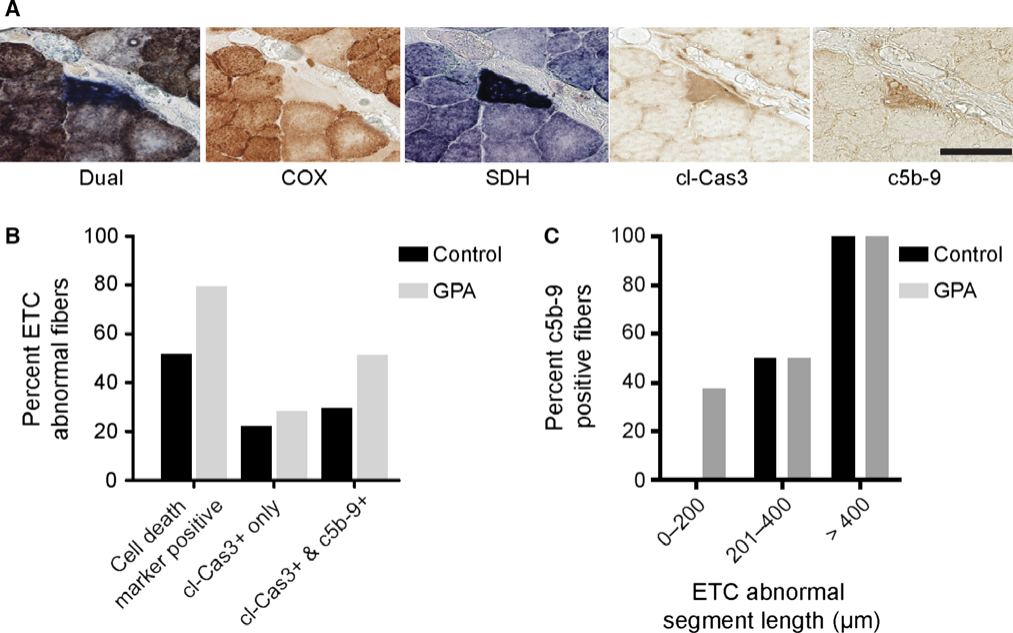
Increased fiber death in GPA‐induced ETC abnormal fibers. (A) Representative ETC abnormal fiber undergoing apoptosis and necrosis in a GPA‐treated rat. Dual and single histochemical staining for COX and SDH. Immunohistochemical staining for cl‐Cas3 and c5b‐9. The scale bar denotes 50 μm. (B) A greater fraction of ETC abnormal fibers are positive for apoptotic (cl‐Cas3) and necrotic (c5b‐9) cell death in GPA‐treated rat muscle as compared to control. (C) Cell death processes are activated in shorter ETC abnormal fiber segments of GPA‐treated rats than in controls.

In aging muscle, ETC abnormal fiber segments initiate apoptosis and necrosis (Cheema *et al*., 2015). If ETC abnormal fibers progress to fiber loss, then the elevated abundance of ETC abnormal fibers following GPA treatment should increase apoptotic and necrotic fibers. Immunohistochemical staining of GPA‐treated and control rat skeletal muscle (Fig. 3A) localized cleaved caspase‐3 (cl‐cas3) and complement‐mediated membrane attack complex component c5b‐9 to ETC abnormal segments. These markers indicate activation of apoptotic and necrotic cell death processes in ETC abnormal fibers. To ascertain the frequency of cell death in ETC abnormal fibers, 66 ETC abnormal fibers were examined from GPA‐treated (39 fibers) and control rats (27 fibers). In total, 80% of ETC abnormal fibers were positive for cell death markers in GPA‐treated rats as compared to 50% in control animals. Most of this difference was accounted for by an increase in the fraction of fibers undergoing necrosis in GPA‐treated rats (Fig. 3B); 20–25% of ETC abnormal fibers stained positive only for cl‐Cas3; however, all c5b‐9 positive fibers were cl‐cas3 positive in GPA‐treated and control rat quadriceps. In addition to an increased incidence of cell death positive ETC abnormal fibers from GPA‐treated rats, GPA treatment also resulted in necrotic ETC abnormal segments whose length was < 200 μm (Fig. 3C). By contrast, in control rats, ETC abnormal fibers underwent necrosis only when > 200 μm in length. A final indicator of cell death in ETC abnormal fiber segments is the presence of broken fibers. In GPA‐treated rats, 23% of ETC abnormal fibers were broken, compared to 15% of the ETC abnormal fibers in control rats. These data suggest an accelerated tempo of deletion mutation accumulation and compression of the time to initiation of cell death by GPA treatment.

**Figure 4:**
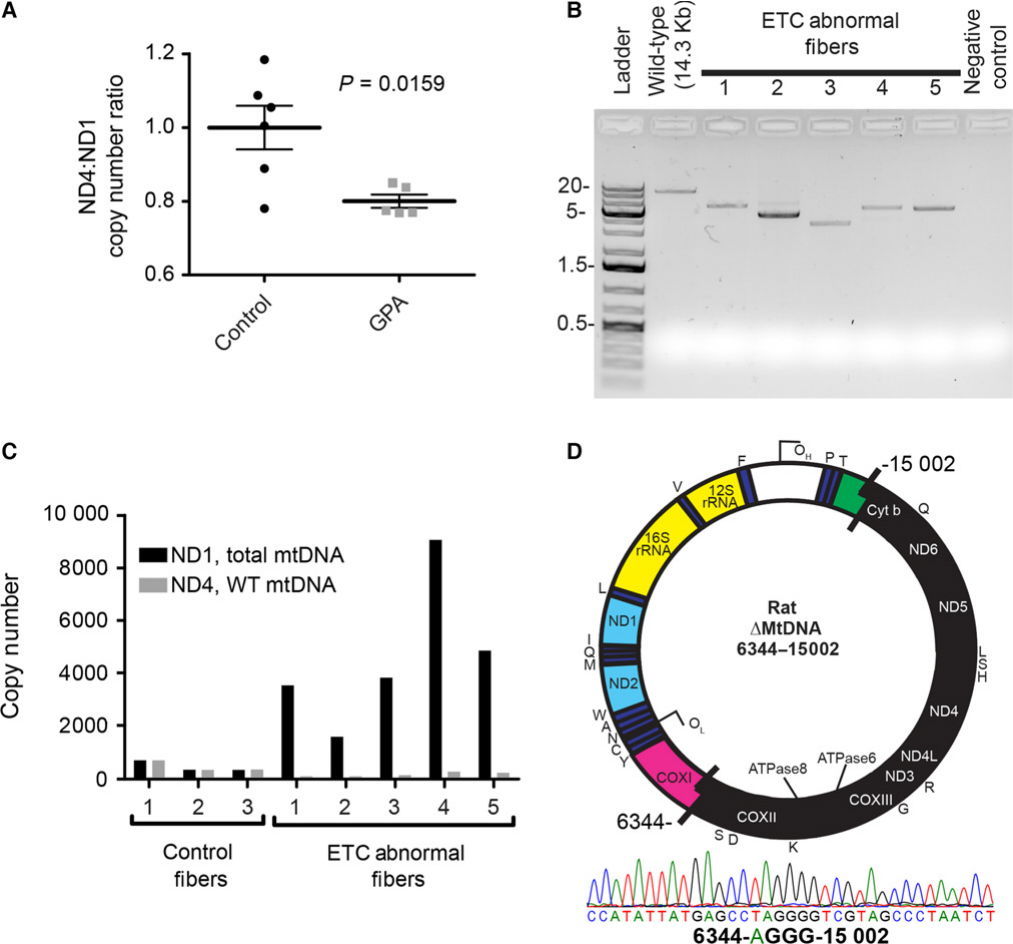
Induction of mtDNA deletion mutation accumulation by GPA treatment in aged rats. (A) Elevated mtDNA deletion mutation frequency in GPA‐treated rat quadriceps muscles. (B) Mitochondrial DNA deletion mutations from GPA‐treated rats amplified by long extension PCR from laser capture microdissected COX‐fibers (C) Copy numbers of total mtDNA as compared to WT mtDNA in single, microdissected control or COX‐fibers showing degrees of heteroplasmy. (D) mtDNA deletion mutation breakpoint sequence from a single microdissected COX‐fiber shown in panels B and C—Fiber 1.

The mtDNA deletion mutations that accumulate with age are large, with 4‐10 kb lost, occur in the major arc and remove up to 11 protein encoding genes and 13 tRNAs. The gene encoding ND4 is routinely deleted (Kowald & Kirkwood, 2014), while minor arc genes such as ND1 are preserved. To quantitate mtDNA deletion burden in muscle homogenates, we exploited differences in copy number between ND4 and ND1 as an estimate of mtDNA deletion mutation frequency (Grady *et al*., 2014) using a digital PCR approach (Taylor *et al*., 2014). In a hypothetical sample comprised of only full length, intact mtDNA molecules, the expected ratio would be 1 and this is what we observed, on average, in tissue homogenate DNA samples from control rats. In GPA‐treated rats however, the ratio was depressed with an average ratio of 0.8 (Fig. 4A) indicating a 20% mtDNA deletion mutation frequency in rat muscle following 4 months of GPA treatment.

While the abundance of deletion mutations in tissue homogenates gives an overall estimation of the mutation frequency, it is the focal distribution of deletion mutations that leads to their cellular impact. Therefore, we coupled laser microdissection and long extension PCR to define the mtDNA genotype within individual COX‐fibers from GPA‐treated rats. Each ETC abnormal fiber contained a unique mtDNA deletion mutation with a loss of 6–10 kb (Fig. 4B). To interrogate heteroplasmy in single COX‐fibers, we quantitated the ND4 and ND1 copy numbers by digital PCR. In COX+ control fibers, we found the expected 1:1 ratio between ND4 and ND1 genes. COX‐fibers, however, possessed large increases in total mtDNA copy number (ND1) but a depression of the ND4 target site encompassed by the deletion event (Fig. 4C). These copy numbers demonstrate > 90% heteroplasmy for mtDNA deletion mutations within single ETC abnormal muscle fibers. DNA sequencing of the LX‐PCR products from one of the COX‐fibers identified a breakpoint between nucleotides 6344 and 15 002 of the F344x BN F1 mtDNA genome (AY769440.1) which included a four base pair repeat. This deletion mutation disrupted 11 protein coding and nine tRNA genes in the major arc while maintaining the minor arc genes including ND1 (Fig. 4D).

## Discussion

The physiological impact of mitochondrial mutations is demonstrated by the effect of AZT treatment (Rayapureddi *et al*., 2010), accelerated aging of mitochondrial DNA polymerase gamma mutator mice (Trifunovic *et al*., 2004), and cardiac arrhythmias in Twinkle mitochondrial helicase mutant mice (Baris *et al*., 2015). Previous experiments demonstrated that a 7 weeks GPA treatment of 27‐month‐old rats resulted in an increased incidence (3.7‐fold) of ETC abnormal fibers, but did not result in measureable fiber loss (Herbst *et al*., 2013). Clonally expanded mtDNA deletion mutations first appear as ETC abnormal fibers at approximately 28 months of age in the hybrid rat (Herbst *et al*., 2013). We hypothesized that inducing mitochondrial biogenesis at older ages, when deletion mutation frequency is higher, would explicitly test the role of these mutations in muscle fiber loss. Four months of GPA treatment in 30‐month‐old rats resulted in a 12‐fold increase in ETC abnormal fibers, accelerating cell death, fiber loss and fibrosis, leading to a 22% loss of muscle mass. This loss encompasses individual fiber loss and atrophy (e.g. due to reduced protein synthesis or increased protein degradation), but is offset by positive contributions to muscle mass from fibrotic replacement. Loss of muscle mass and rectus femoris fiber number in control rats was consistent with previous data (Lushaj *et al*., 2008). The sparing of muscle deterioration in the central areas of the quadriceps follows from the muscle fiber type distribution (predominantly type I) in these regions. Type II fibers, which predominate in the muscle periphery, have been shown to be more susceptible to deletion mutation accumulation and are preferentially lost with age (Wanagat *et al*., 2001; Bua *et al*., 2002). GPA treatment, however, could also be acting in these areas independent of mitochondrial genotype as GPA is known to cause a type I to type II fiber shift (Oudman *et al*., 2013). Increased deposition of fibrotic tissue in GPA‐treated rat quadriceps muscle is consistent with the inflammatory loss of necrotic ETC‐deficient muscle fibers.

In muscle aging, activation of apoptosis and necrosis predominantly occurs in ETC abnormal muscle fibers (Cheema *et al*., 2015). The ETC abnormality results from the focal accumulation of mtDNA deletion mutations (Wanagat *et al*., 2001; Herbst *et al*., 2007). As GPA treatment promotes sarcopenic changes through an increase in ETC abnormal fiber abundance, treatment should also accelerate the mitochondrial genotypic changes observed with muscle aging. To test this relationship, we quantitated mtDNA deletion mutation abundances in both muscle tissue homogenates and single muscle fibers. The mtDNA deletion frequency (20%) in tissue homogenates mirrored the increased abundance of ETC abnormal fibers. Similarly, in single fibers, GPA treatment resulted in deletion mutation abundances that exceed the 90% phenotypic threshold for presentation of a respiration deficiency. The mtDNA deletions are clonal, involve only the major arc, and ranged in size from 4 to 10 kb. The deletion mutations induced by GPA treatment appear similar to those observed previously (Cao *et al*., 2001; Wanagat *et al*., 2001). Previously, in rats, we observed that the abundance of wild‐type genomes was maintained in ETC abnormal regions (Herbst *et al*., 2007). Subsequent data in humans showed a diminished abundance of wild‐type genomes (Herbst *et al*., 2007). The data presented here are more similar to the latter, a slight reduction in wild‐type mtDNA abundance.

The approach to quantitation of ETC abnormal fibers and mitochondrial deletion mutations depends upon the underlying abundance (Greaves *et al*., 2014). The abundance of mtDNA deletion mutations in normally aged muscle contrasts dramatically with abundances following GPA treatment illustrating the need for different approaches to quantitation. Determining ETC abnormal fiber number in normally aged muscle requires counting all lesions within a given tissue volume to account for the three‐dimensional structure, distribution, and abundances of these lesions (Wanagat *et al*., 2001). In contrast, the elevated abundance of ETC abnormal fibers following GPA treatment required a sampling approach; volume density techniques were no longer practical due to the massive increase in ETC abnormal fiber number. Detection of deletion mutation events from tissue homogenates during normal aging is exceedingly difficult due to the large range between deletion mutation‐containing and wild‐type mtDNA genome abundances. Deletion mutation‐containing mitochondrial genomes have been quantitated at between 20 parts per million in human brain homogenates (Taylor *et al*., 2014) and one part per million in aged mouse muscle (Blalock *et al*., 2010) vs. 20 parts per hundred in homogenates from GPA‐treated rats and > 90 parts per hundred in single ETC abnormal fibers. The absolute mutation frequency in aged control rats is below the detection range using the loss of the major arc primer/probe site quantitation method, which results in a ND4:ND1 ratio approximating one. The higher deletion mutation abundance in homogenates from GPA‐treated rat muscles or single muscle fibers allowed detection of deletion mutation frequencies ranging from 20% in homogenates to 98% in single fibers.

The induction of ETC abnormal fibers by GPA treatment suggested two possibilities: (i) GPA treatment generated mtDNA deletion mutations *de novo* or (ii) GPA enhanced the accumulation of latent preexisting, age‐induced deletion mutations. GPA is not known to be mutagenic despite numerous studies of its activity in mammals (reviewed by Oudman) (Oudman *et al*., 2013; Karamat *et al*., 2015), and we did not observe any ETC abnormalities in young rats treated with GPA (Herbst *et al*., 2013). GPA inhibits creatine‐dependent energy metabolism leading to fiber atrophy in young and old rodents and induces mitochondrial biogenesis in skeletal muscle (Mahanna *et al*., 1980; Wiesner *et al*., 1999; Zong *et al*., 2002; Herbst *et al*., 2013). Sampling individual respiration competent fibers determined that 10–25% of these ETC normal fibers from 36‐month‐old rat skeletal muscles harbored mtDNA deletion mutations at intrafiber abundances of 0.01–2.17% (Pak & Aiken, 2004). These latent mutations are insufficient to cause a loss of cytochrome c oxidase activity within the fiber. Clonally expanded mtDNA deletion mutations, within respiratory‐deficient fibers, are the conspicuous portion of the mutation population (Herbst *et al*., 2007), while latent, unexpanded mtDNA deletion mutations represent a reservoir for future clonal expansion and fiber loss. Our studies suggest that GPA treatment reveals these latent mtDNA deletion mutations by driving their clonal accumulation, phenotypic expression, and fiber loss within a 4‐month period. This informs our understanding of the natural progression of mitochondrial deletion mutations in aging—given sufficient time or stimulus, latent deletion mutations will manifest in future fiber loss.

The increased incidence of respiratory‐deficient muscle fibers by expansion of latent mtDNA deletion mutations demonstrates the existence of specific pathways that control mitochondrial DNA mutation accumulation and, more importantly, that these pathways can be modulated *in vivo*. While the activation of mitochondrial biogenesis is suspected to have beneficial effects on aging (Martin‐Montalvo *et al*., 2013; Yang *et al*., 2015), if that process lacks selectivity for competent mitochondrial genomes, adverse effects may accrue due to the expansion of mtDNA deletion mutations in mammals (Lin *et al*., 2016). Similarly, treatments designed to affect aging phenotypes by manipulating mitochondrial quality control require examination of those interventions on both normal and mutant mtDNA populations.

GPA treatment in aged rats increased the accumulation of mtDNA deletion mutations, which manifests as an increased abundance of ETC abnormal muscle fibers. In addition to increasing the abundance of ETC abnormal segments, GPA accelerated the progression of ETC abnormal fiber segments as evidenced by shorter abnormalities, increased cell death activation and cell death activation in shorter segments, and increased incidence of broken fibers. Finally, GPA treatment accelerated the loss of muscle mass and fiber number that defines sarcopenia. The experimental manipulation of mtDNA deletion mutation abundance by a pharmacological intervention in old animals accelerated phenotypes of muscle aging. These data strengthen the causal link between mtDNA deletion mutation and fiber loss and underscore the significance of latent mtDNA deletion mutations. The exogenous pharmacological induction of ETC abnormalities implicates specific pathways that regulate mtDNA deletion mutation accumulation *in vivo*. Modulation of these pathways is likely to be pleiotropic with beneficial effects on bioenergetics confounded by the antagonistic induction of mutant mtDNA accumulation.

## Experimental procedures

### Ethics statement

This study was carried out in accordance with the recommendations in the NIH Guide for Care and Use of Laboratory Animals and the guidelines of the Canadian Council on Animal Care. The protocols used were approved by the Institutional Animal Care and Use Committees at the University of Alberta.

### Animals, GPA treatments, and tissue preparation

Thirty‐month‐old male Fischer 344 x Brown Norway F1 hybrid rats (*Rattus norvegicus*) were purchased from the National Institute on Aging colony maintained by Harlan Sprague Dawley (Indianapolis, IN, USA). β‐guanidinopropionic acid was synthesized as described (Herbst *et al*., 2013)] from cyanamide and β‐alanine. GPA was purified by recrystallization and the synthesis confirmed by mass spectrometry and IR spectroscopy. β‐GPA was formulated to 1% by weight in 6% fat rodent chow (Harlan‐Teklad, Madison, WI, USA) and fed for 4 months *ad libitum*. Rats were housed on a 12‐h light/dark cycle. No significant difference was observed in the survival of rats treated with β‐GPA vs controls. Animals were euthanized, the quadriceps muscles dissected from the animals, weighed, bisected at the mid belly, embedded in optimal cutting temperature compound (Sakura Finetek, Torrance, CA, USA), flash frozen in liquid nitrogen, and stored at −80 °C. A minimum of one hundred 10‐μm‐thick consecutive transverse cross sections were cut with a cryostat at −20 °C and placed on Probe‐On‐Plus slides or PEN membrane glass slides (Life Technologies, Carlsbad, CA, USA) for laser microdissection. Slides were stored at −80 °C until needed.

### Histochemical and immunohistochemical staining

At 70‐um intervals, sections were stained for COX (brown) or SDH (blue) as previously described (Wanagat *et al*., 2001 p. 1409). A third slide was dual stained, first for COX and secondly for SDH. ETC abnormal fibers appear blue on a brown background following dual staining. Two slides within the series were used for hematoxylin and eosin (H&E) staining. Fibrotic tissue was stained using Masson's trichrome, as described (Lushaj *et al*., 2008). Immunohistochemical staining using anti‐activated caspase‐3 (Promega, Madison, WI, USA; 1:200) and anti‐C5b‐9 (Abcam, Cambridge, MA, USA; 1:500) was performed as described (Cheema *et al*., 2015). After histochemistry or immunohistochemistry, slides were digitized by a scanning microscope (Nanozoomer, Hamamatsu).

### Image analysis, counts, and quantitation

Cross‐sectional area measurements and fiber counts of the rectus femoris were obtained from digital images. Rectus femoris muscle is used for CSA measurements and fiber counts because it is entirely encapsulated by the quadriceps and has low pennation angle to the muscle fibers, facilitating accurate and precise quantification. The absolute number of ETC abnormal fibers (COX‐/SDH++) was identified and annotated throughout 10 μm of tissue in both GPA‐treated and control rats as reported in Fig. 2. To estimate the volume density of ETC abnormal fibers, 119 individual randomly selected fibers from GPA‐treated rats were followed throughout one millimeter of tissue to measure the abundance of ETC abnormal regions. Fibers that stained positive for cleaved caspase‐3 or C5b‐9 at a 100‐μm interval were counted, annotated, and followed throughout the 1 mm of rectus femoris tissue. The length of ETC abnormality segments was determined by counting the number of slides the abnormality appeared in and multiplying by 10 μm.

### DNA isolation

Rat quadriceps muscle was ground to a powder using a mortar, pestle, and liquid nitrogen. Total DNA was extracted using the Maxwell 16 tissue preparation kit (Promega). Total DNA quantity and quality was measured using spectrophotometry at A230, A260, and A280 (Thermo Scientific Nanodrop 2000 Spectrophotometer, Carlsbad, CA, USA) and integrity examined by gel electrophoresis.

DNA isolation from individual muscle fiber cross sections was performed subsequent to laser microdissection. Muscle sections on membrane slides were dehydrated in absolute ethanol and air‐dried. ETC abnormal fibers were identified on adjacent serial sections by staining for COX and SDH as described above. Single muscle fiber sections were captured using a Leica LMD 7000, digested in 1 μL of proteinase K digestion solution as described (Wanagat *et al*., 2001; Herbst *et al*., 2007), and re‐suspended to a total volume of 10 μL with nuclease‐free water. Initial estimation of mtDNA copy number from all DNA samples was obtained by quantitative PCR (qPCR) using primers and probes (Table S1, Supporting information) to the rat mitochondrial genome (ND4, ND1) purchased from Integrated DNA Technologies (IDT; Coralville, Iowa). qPCRs for both ND4 and ND1 were 1 μL of target DNA, 1.25 μL of 20X ND4/ND1 primer probe mix, 12.5 μL of 2X AmpliTaq Master Mix (ThermoFisher; Waltham, MA), and 10.25 μL of H_2_O for each 25 μL reaction. qPCR cycling conditions for ND4 were Taq polymerase activation at 95 °C for 10 min, denaturation at 95 °C for 10 s, annealing at 58 °C for 10 s, extension at 72 °C for 30 s, plate read, and repeat steps 2–5, for a total of 40 cycles. ND1 was cycled similarly, except the annealing temperature was decreased to 54 °C.

### Digital PCR quantitation of mtDNA deletion mutation abundance

Samples were diluted to the target range of the Quantstudio 3D digital PCR (dPCR) 20K Chip (Version 2; ThermoFisher, Waltham, MA, USA) and the mtDNA copy number determined in each sample. ND4 and ND1 reactions were performed on separate, individual digital PCR chips according to the manufacturer's instructions. DPCR cycling conditions for ND4 and ND1 were the same as for qPCR described above. MtDNA copy numbers per microliter were calculated using QuantStudio 3D Analysis Suite Cloud Software (Version 3, ThermoFisher). For the tissue homogenates, mtDNA copy numbers were normalized to the control samples.

### Long extension PCR amplification of mtDNA deletions from single muscle fibers

Deletion‐containing mtDNAs were amplified using the 16S‐F and 12S‐R primer set (Table S1, Supporting information) purchased from IDT. Long extension PCRs were assembled according to the manufacturer's instructions, Go‐Taq Long PCR master mix (Promega). PCR cycling conditions were polymerase activation at 95 °C for 2 min, denaturation at 94 °C for 20 s, and annealing at 68 °C for 10 min, repeated for 40 cycles. PCR products were fractionated on 1% agarose gels, stained with ethidium bromide, and visualized under UV light.

### Statistical analysis

Data are presented as mean ± SEM. Reported *P*‐values were obtained using Student's *t*‐tests with a *P*‐value < 0.05 being significant. Summary statistics for all data are presented in Tables S2 and S3 (Supporting information). All statistical analyses were performed using GraphPad Prism version 5.00 for Windows (GraphPad Software, San Diego, CA, USA).

## Funding

This work was supported by the American Federation for Aging Research (JW), the Glenn Foundation for Medical Research (JW), the UCLA Hartford Center of Excellence (JW), National Institute on Aging Grants K08 AG032873 (JW), R01 AG030423 (JMA), UCLA Older Americans Independence Center P30 AG028748 (JW), Ellison Medical Foundation New Scholar Award (JW) and Senior Scholar Award (JMA), UCSD/UCLA Diabetes Research Center Pilot and Feasibility Grant (JW).

## Author contributions

AH, JW, NC, DM, and JMA conceived and designed the experiments. AH, JW, KW, and NC performed the experiments. AH, JW, KW, and NC analyzed the data. AH, JW, NC, DM, and JMA wrote the article.

## Conflict of interest

The authors report no conflict of interests.

## Supporting information


**Table S1** Nucleic acid primers and probes described in the manuscript.
**Table S2** Measurements from 34‐month old F344xBN F1 hybrid rats.
**Table S3** MtDNA copy numbers from control and ETC abnormal skeletal muscle fiber cross‐sections from GPA treated rats.
