# Supplementary material for: Latent mitochondrial DNA deletion mutations drive muscle fiber loss at old age
Source: Aging Cell. 2016 Aug 25;15(6):1132–9. doi: 10.1111/acel.12520 (PMC5114866; doi:10.1111/acel.12520)
Supplement: Supplementary file 1 — Table S1 Nucleic acid primers and probes described in the manuscript. Table S2 Measurements from 34‐month old F344xBN F1 hybrid rats. Table S3 MtDNA copy numbers from control and ETC abnormal skeletal muscle fiber cross‐sections from GPA treated rats. [file ACEL-15-1132-s001.docx]

Supporting Table 1. Nucleic acid primers and probes described in the manuscript.

| Primer/Probe Location | Sequence |
| --- | --- |
| ND4-F | ATGGGAGCTACAATACTAATAATC |
| ND4-P | FAM/CCACGGCTT/ZEN/AACCTCCTCAC |
| ND4-R | CTTGCTAATAGTCATCATGTT |
| ND1-F | GTCACAATAGCCATTATCCTCTTA |
| ND1-P | HEX/TCCTCCTAA/ZEN/TAAGCGGCTCCT |
| ND1-R | GGGATTAATAGTCAGATATTCTG |
| 16S-F | GGGTTTACGACCTCGATGTTGGATCAGGACATCCCAAT |
| 12S-R | ACCGCGGTGGCTGGCACGAAATTTACCAACCCTGA |

Supporting Table 2. Measurements from 34-month old F344xBN F1 hybrid rats.

|  | Ctrl  (Mean ± SE) | GPA  (Mean ± SE) | p-value |
| --- | --- | --- | --- |
| Body Mass (g) | 515.3 ± 23.95 | 402.1 ± 18.57 | 0.0022 |
| Quadriceps Mass (g) | 4.92 ± 0.186 | 3.82 ± 0.266 | 0.0042 |
| Fiber number | 8859 ± 162.2 | 7270 ± 323.6 | 0.0023 |
| RF CSA (mm^2^) | 34.0 ± 1.77 | 26.8 ± 2.3 | 0.0377 |
| Fiber CSA (um^2^) | 2,853 ± 110.7 | 1,517 ± 34.30 | 0.0001 |
| % Collagen | 4.33 ± 0.89 | 24.42 ± 4.67 | 0.0018 |
| Brain Mass (g) | 2.26 ± 0.069 | 2.32 ± 0.041 | 0.2144 |
| Heart Mass (g) | 1.41 ± 0.022 | 1.19 ± 0.174 | 0.2699 |
| ETC Abnormality Abundance in Quadriceps | 12.3 ± 3.0 | 154.0 ± 26.3 | 0.0090 |
| Mean ETC Abnormality Segment Length (um) | 442 ± 49.0 | 345 ± 19.4 | 0.0384 |
| Homogenate WT/Total mtDNA ratio* (ND4/ND1) | 1.0 ± 0.054 | 0.80 ± 0.114 | 0.0159 |

*Ratios normalized to control samples.

Supporting Table 3. MtDNA copy numbers from control and ETC abnormal skeletal muscle fiber cross-sections from GPA treated rats.

|  | Ctrl  (Mean ± SE) | ETC abnormal (Mean ± SE) |
| --- | --- | --- |
| ND1 copies/fiber* (Total mtDNA) | 4,225 ± 1,199 | 45,627 ± 12,448 |
| ND4 copies/fiber*  (WT mtDNA) | 4,230 ± 1,185 | 1,245 ± 365 |
| % Heteroplasmy | 0.00 | 97.2 |
